# How do sustainable diets fit into the climate agenda?

**DOI:** 10.1186/s40985-016-0034-3

**Published:** 2016-11-11

**Authors:** Lukasz Aleksandrowicz

**Affiliations:** 1 London School of Hygiene & Tropical Medicine (LSHTM), London, UK; 2 Leverhulme Centre for Integrative Research on Agriculture and Health (LCIRAH), London, UK

**Keywords:** Food consumption, Greenhouse gas emissions, Water and land use, Non-communicable diseases, Diet

## Abstract

Food production is a major driver of greenhouse gas (GHG) emission and other environmental footprints, and dietary risk factors are contributors to non-communicable diseases. A growing body of evidence has shown that changes in what and how much we eat can offer benefits for both the environment and health. However, several data gaps and complexities remain in this research area. A better understanding and increased uptake of sustainable diets will require further research, investment, and interdisciplinary collaboration.

## Background

When the public thinks of major sources of greenhouse gas (GHG) emissions, agriculture does not seem to be at the forefront of their minds [1]. However, agriculture contributes about one quarter of all emissions, a magnitude comparable with other major sectors, including energy production (35% of global emissions), industry (21%), and transport (14%) [2]. Efforts to reduce GHG emissions require action across all sectors, and therefore, agriculture will have to implement its own mitigation solutions. Beyond GHG emissions, food production is also responsible for about 70% of global water use and takes up one third of potentially cultivatable land [3].

## Main text

Mitigation of GHG emissions is possible in various areas of food production and consumption, and approaches are broadly classified as supply side (technical innovations producers can achieve) and the demand side (how much and which foods consumers choose to eat) efforts. Action will be needed in both spheres, though evidence suggests that opportunities may be larger on the consumer side [4].

This raises the question of what food choices consumers can make to limit GHG emissions. The literature has shown that different foods can have markedly varied levels of emissions, with ruminant meat generally showing the highest emissions per calorie, followed by other meats and dairy, and plant-based foods having the least emissions [5]. Studies from high-income countries, where average diets tend to be high in animal-based foods and overall calories, show that health and climate benefits can be achieved by replacing meat and dairy intake with plant-based foods [6]. Additional benefits from these shifts can also be realized in land and water use. Many of these benefits can be achieved by following national dietary guidelines.

However, studies also point to complexities in these relationships. Some foods that should be restricted in our diets may have relatively low emissions, such as sugar. Foods that have low GHG emissions may have relatively more detrimental effects on other environmental indicators such as water use [7]. The opportunities for win-win strategies in environment and health are also unclear in low-income countries, where data on environmental impacts of food production are scarce and where many individuals may need to consume more, rather than fewer, calories and increase their diversity of food intake.

## Conclusion

Ultimately, more needs to be done to comprehensively evaluate the impacts of shifting to low GHG diets. Further work should focus on strengthening the many gaps in region- and item-specific GHG emissions of food production and value chains. A broader assessment of sustainability will also require measurement of dietary shifts against a wider set of environmental, health, economic, and socio-ethical indicators. These efforts will require sustained investment in this emerging research area and interdisciplinary collaboration.

However, despite these gaps, there is evidence that diets can play an important role in mitigation of GHG emissions. Climate and health benefits can be currently achieved in many regions by at least partial replacement of high intake of animal-based foods (particularly ruminant meat), with intake of plant-based foods (including an appropriate mix of pulses, cereals, fruit and vegetables).

## Abbreviation

GHG: greenhouse gas
